# Supplementary material for: FLTX2: A Novel Tamoxifen Derivative Endowed with Antiestrogenic, Fluorescent, and Photosensitizer Properties
Source: Int J Mol Sci. 2021 May 19;22(10):5339. doi: 10.3390/ijms22105339 (PMC8161337; doi:10.3390/ijms22105339)

Supplementary Figure S1. ^{1}H NMR spectra of compound **2**.

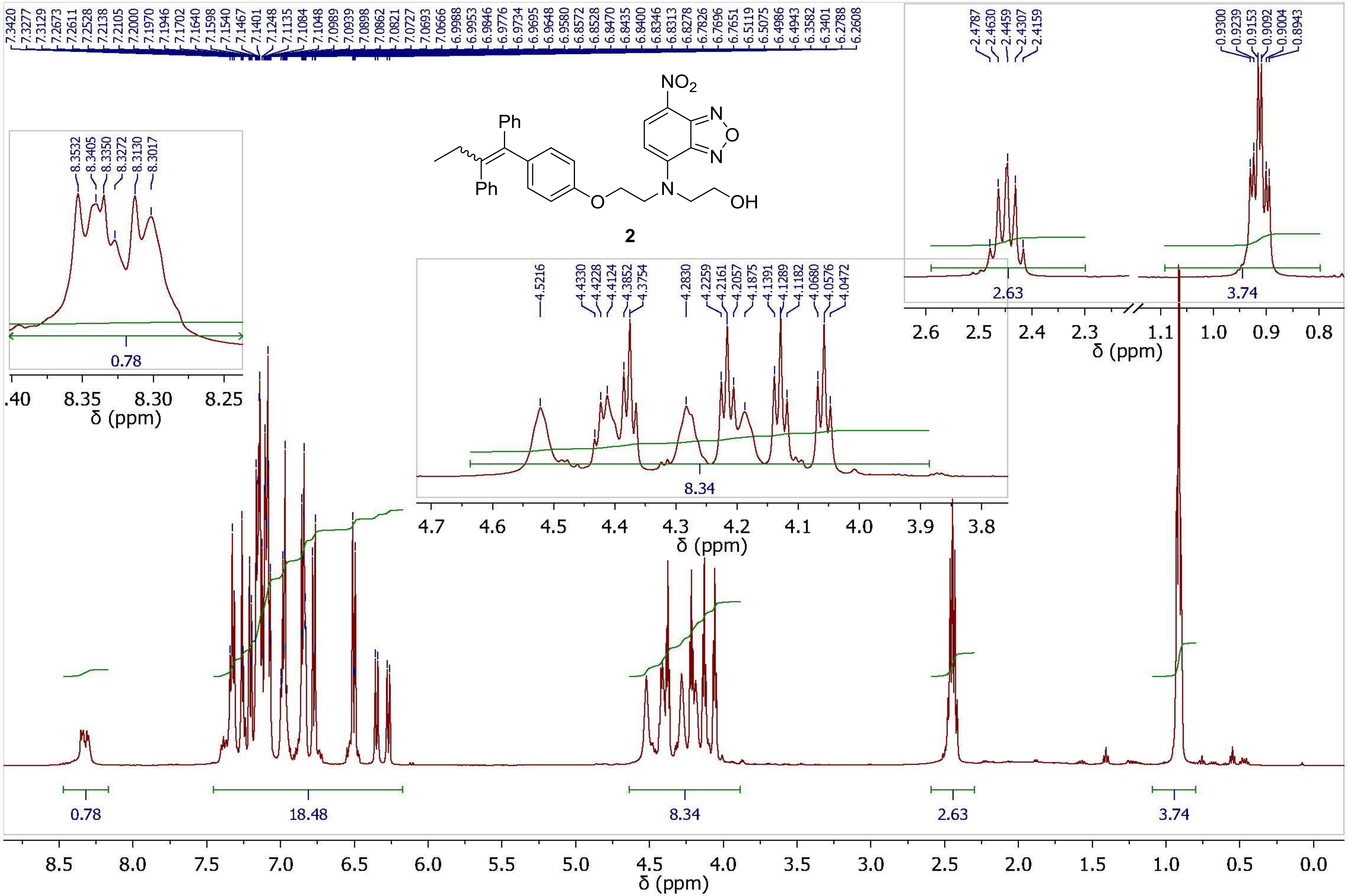

Supplementary Figure S2. ^{13}C NMR spectra of compound **2**.

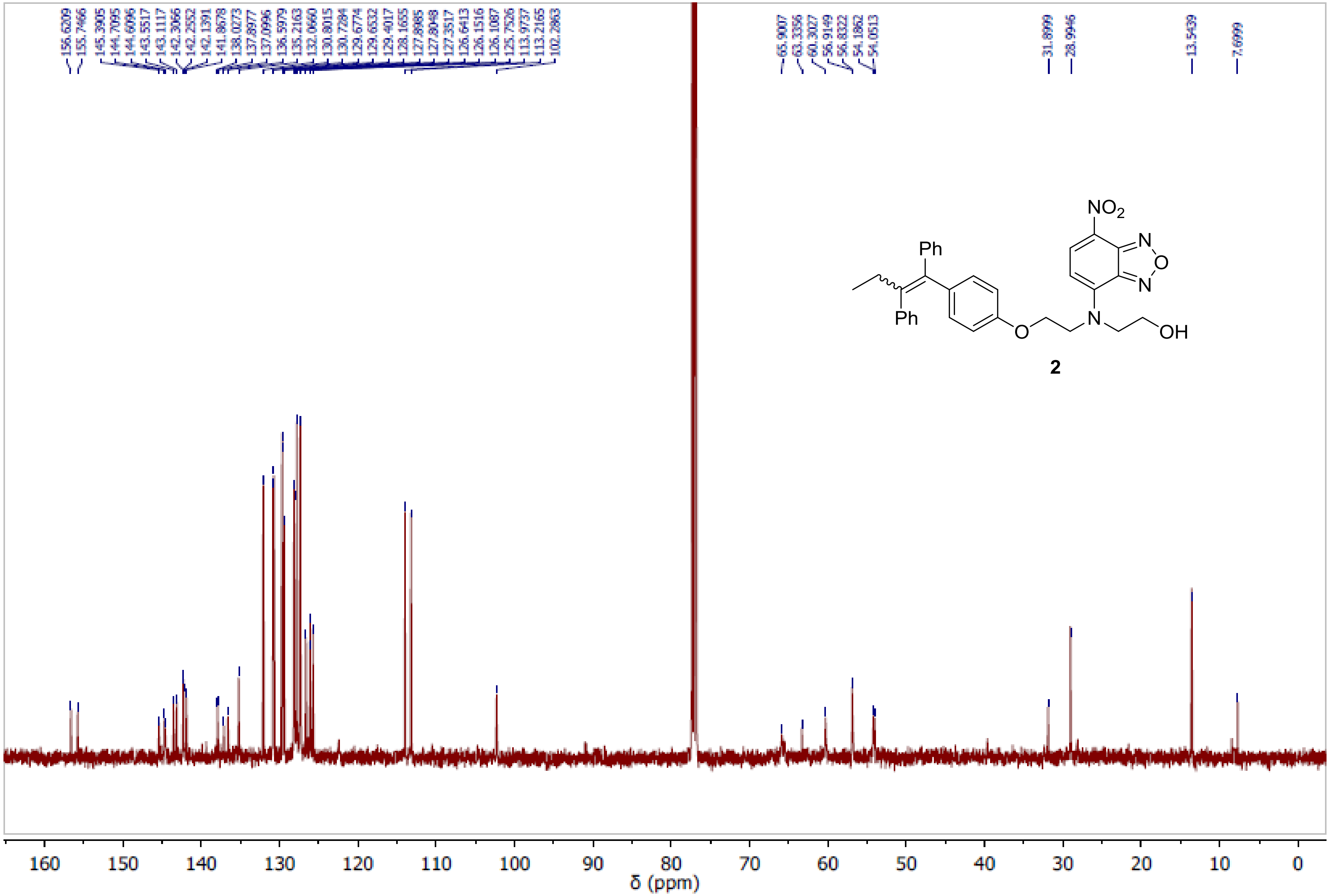

Supplementary Figure S3. ^{1}H NMR spectra of compound **3**.

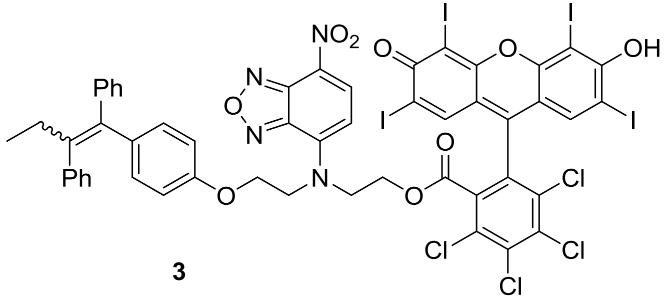

3

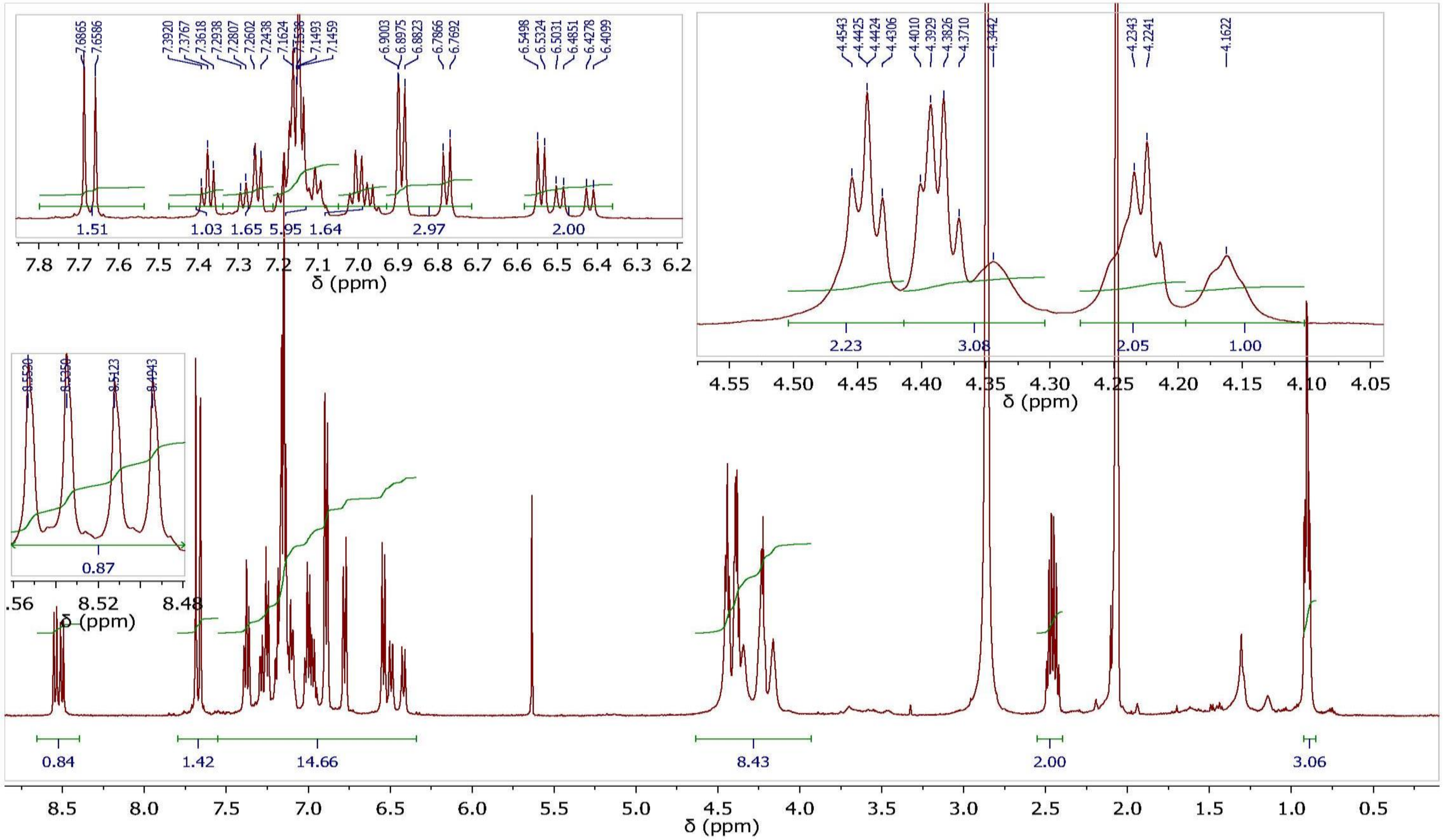

Supplementary Figure S4. ^{13}C NMR spectra of compound **3**.

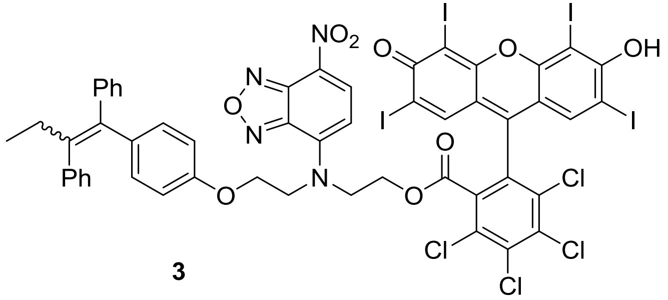

3

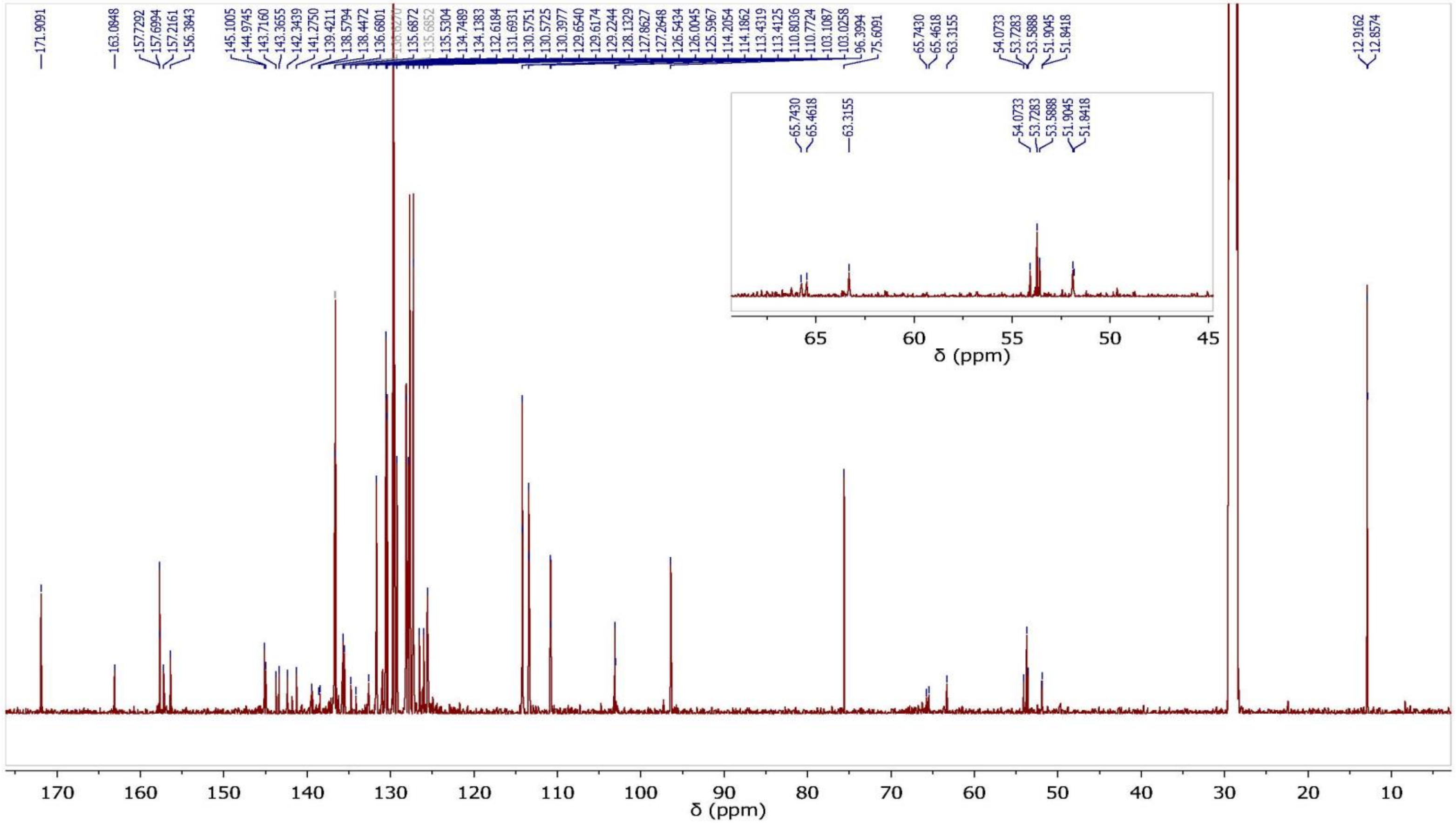

Supplementary Figure S5. DEPT

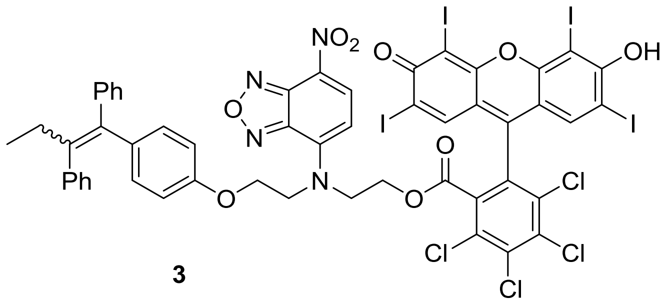

3

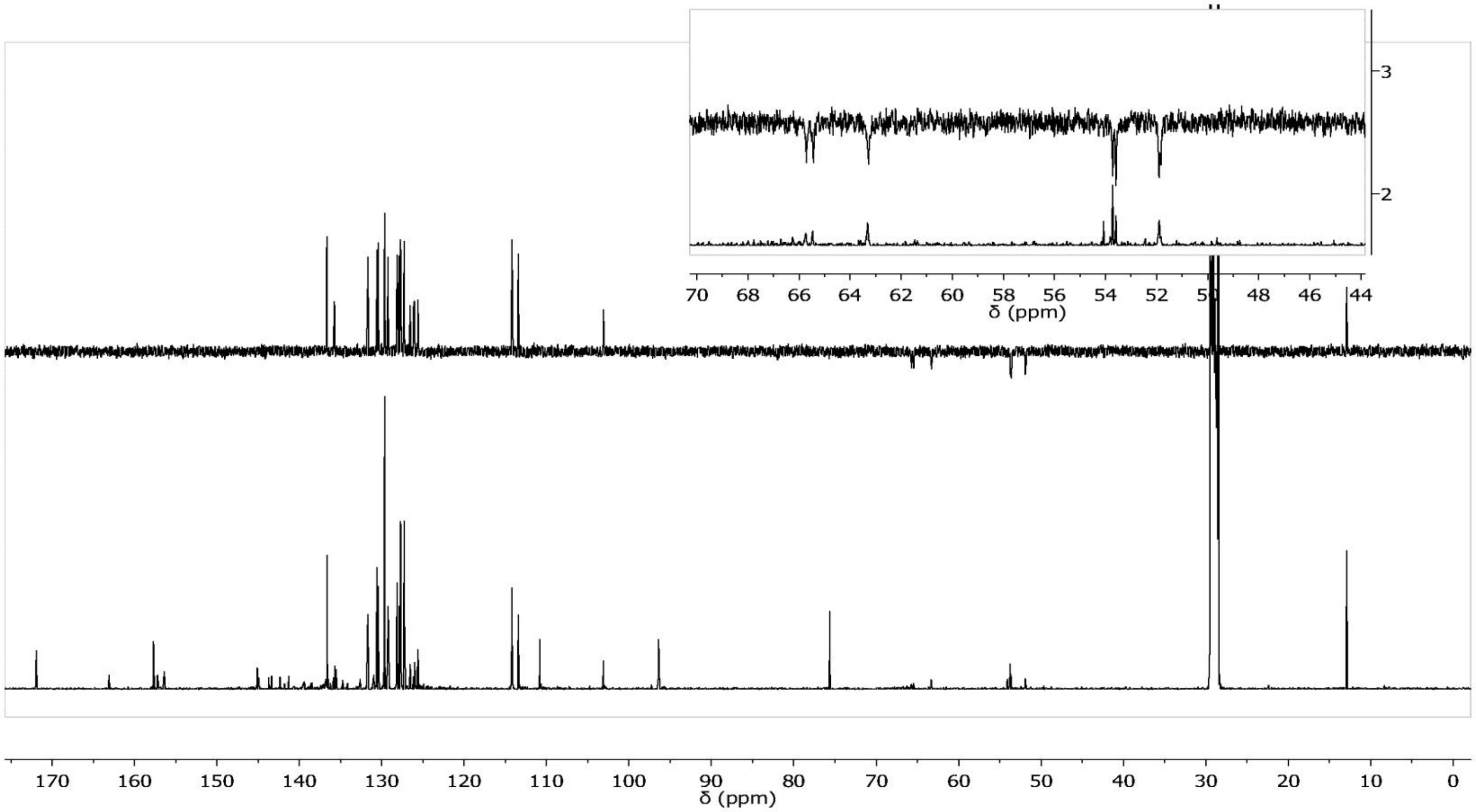

Supplementary Figure S6. HSQC

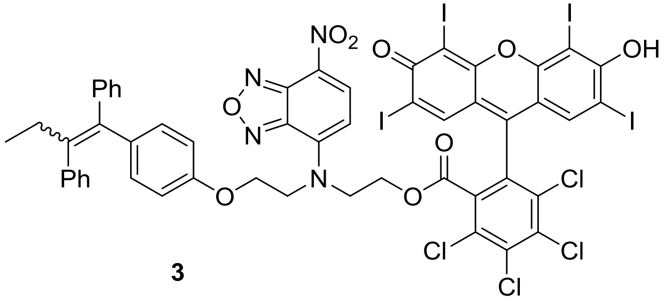

3

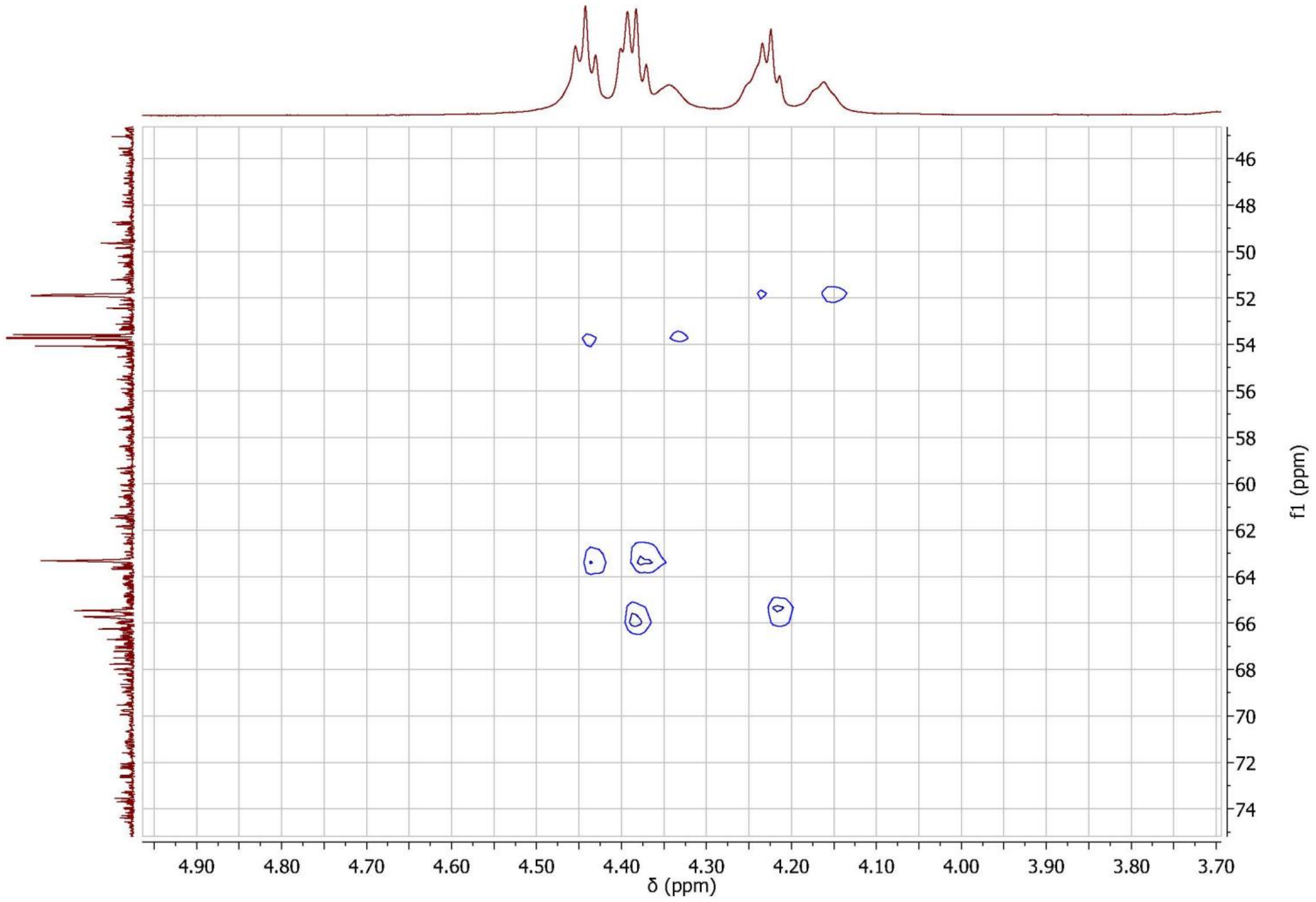

Supplementary Figure S7. Molecular Dynamics study for FLTX2 on the human ERα LBD: Interactions diagram.

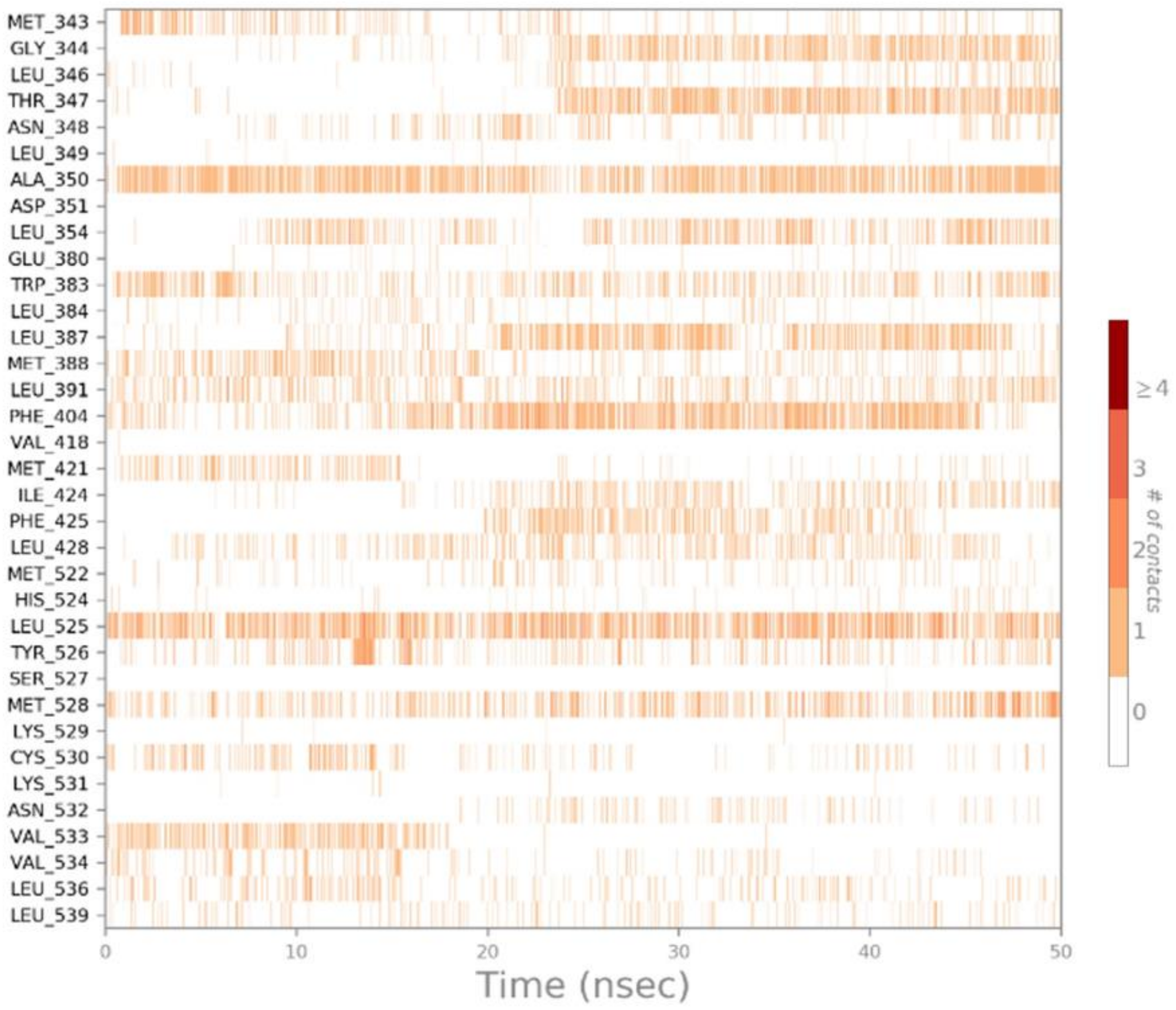

Supplement: Supplementary file 1 [file ijms-22-05339-s001.zip › ijms-1179152-supplementary.pdf]
